# Explainable Deep Learning Framework for Reliable Species-Level Classification Within the Genera *Desmodesmus* and *Tetradesmus*

**DOI:** 10.3390/biology15010099

**Published:** 2026-01-03

**Authors:** İlknur Meriç Turgut, Dilara Gerdan Koc, Özden Fakıoğlu

**Affiliations:** 1 Department of Fisheries and Aquaculture Engineering, Faculty of Agriculture, Ankara University, 06110 Ankara, Türkiye; 2 Department of Agricultural Machinery and Technologies Engineering, Faculty of Agriculture, Ankara University, 06110 Ankara, Türkiye; dgerdan@ankara.edu.tr; 3 Department of Basic Science, Faculty of Fisheries, Ataturk University, 25240 Erzurum, Türkiye; ozden.fakioglu@atauni.edu.tr

**Keywords:** explainable artificial intelligence (XAI), deep learning, saliency map, Grad-CAM, model interpretability, taxonomic resolution, phytoplankton

## Abstract

Microalgae are an evolutionarily ancient and morphologically diverse group of photosynthetic eukaryotes, with taxonomic resolution complicated by environmentally driven phenotypic plasticity. This study merges deep learning and explainable artificial intelligence (XAI) to establish a transparent, reliable, and biologically meaningful framework for green microalgae (Chlorophyta) classification. Microscope images from three morphologically distinct algal species—*Desmodesmus flavescens*, *Desmodesmus subspicatus*, and *Tetradesmus dimorphus* representing the genera *Desmodesmus* and *Tetradesmus* within Chlorophyta—were analyzed using twelve convolutional neural networks, including EfficientNet-B0–B7, DenseNet201, NASNetLarge, Xception, and ResNet152V2. A curated dataset comprising 3624 microscopic images from three Chlorophyta species was used, split into training, validation, and test subsets. All models were trained using standardized preprocessing and data augmentation procedures, including grayscale conversion, CLAHE-based contrast enhancement, rotation, flipping, and brightness normalization. The model’s performance was assessed using accuracy and loss metrics on independent test datasets, while interpretability was evaluated through saliency maps and Gradient-weighted Class Activation Mapping (Grad-CAM) visualizations. ResNet152V2 achieved the highest overall performance among all evaluated architectures, outperforming EfficientNet variants, NASNetLarge, and Xception in terms of macro F1-score. Visualization analysis showed that both Grad-CAM and saliency mapping consistently highlighted biologically relevant regions—including cell walls, surface ornamentation, and colony structures—confirming that the models relied on taxonomically meaningful features rather than background artifacts. The findings indicate that the integration of deep learning and XAI can attain consistently high test accuracy for microalgal species, even with constrained datasets. This approach enables automated taxonomy and supports biodiversity monitoring, ecological assessment, biomass optimization, and biodiesel production by integrating interpretability with high predictive accuracy.

## Simple Summary

The accurate identification of microalgae remains challenging due to imaging variability, physiological changes, and environmental factors rather than inherent morphological similarity. This study presents an interpretable artificial intelligence framework designed to classify green algae species from microscope images with both high accuracy and transparency. Twelve deep learning models were systematically trained and compared using standardized image processing on three distinct species—*Desmodesmus flavescens*, *Desmodesmus subspicatus* and *Tetradesmus dimorphus*. Among the evaluated architectures, several convolutional neural networks achieved strong performance, with macro-level precision, recall, and F1-scores generally exceeding 0.90, and the best-performing model (ResNet152V2) reaching a macro F1-score of 0.975. Visualization analyses confirmed that the models based their decisions on true biological features, such as cell walls and surface structures, rather than irrelevant background elements. These findings demonstrate that artificial intelligence can achieve near-perfect recognition of microalgae even from limited datasets while maintaining interpretability. The proposed framework provides a reproducible and biologically meaningful tool for digital taxonomy.

## 1. Introduction

Spanning multiple evolutionary lineages, algae encompass a spectrum of cellular architectures and adaptive traits that complicate accurate and reproducible taxonomic resolution. Acting as primary producers, they sustain food webs, regulate nutrient fluxes, and contribute substantially to atmospheric oxygen generation. Their ecological and economic significance—ranging from environmental monitoring and aquaculture to biotechnology and renewable energy—further heightens the need for precise and reliable species identification. In this context, the inherent structural complexity of algal taxa underscores the demand for analytical approaches capable of capturing subtle morphological variation and distinguishing closely related groups with greater consistency. Classical approaches based on morphometric traits are limited by subjective interpretation, time-intensive procedures, and morphological plasticity influenced by environmental variables such as light, nutrient availability, and physiological stress [1]. This phenotypic variability frequently results in misclassification, especially among closely related microalgae whose diagnostic characters can partially overlap. Although molecular approaches have greatly advanced phytoplankton systematics, they have also revealed a pronounced incongruence between morphology and phylogeny in unicellular green microalgae. As highlighted by Krienitz and Bock [2], multiple phylogenetic species may share identical morphotypes, while morphologically variable taxa often comprise genetically distinct lineages, resulting in widespread polyphyly among traditional genera. This challenge is particularly evident within Scenedesmaceae, where extensive morphological plasticity in genera such as *Scenedesmus* complicates reliable species-level identification under light microscopy. Consequently, morphology-based taxonomy remains both indispensable and inherently problematic, especially in routine ecological monitoring.

In recent years, the advent of artificial intelligence and, more specifically, deep learning, has revolutionized biological image analysis, enabling automated, scalable, and highly accurate recognition of complex morphological structures. Deep learning models, particularly convolutional neural networks (CNNs) and transformer-based architectures, have demonstrated exceptional performance in taxonomic classification tasks, achieving accuracies frequently exceeding 90% and occasionally surpassing 99%. Foundational CNNs such as AlexNet and VGG have been instrumental in pioneering this field, as evidenced by Pedraza et al. [3], who achieved 99% accuracy in the classification of eighty diatoms. Successive developments, including deep residual networks such as ResNet [3,4,5,6,7], introduced hierarchical feature extraction and improved gradient propagation, yielding greater stability and interpretability. EfficientNet architectures, designed for compound scaling, have achieved remarkable precision even with limited training data [8,9], while compact models such as MobileNet [10,11,12] have proven suitable for real-time and low-resource applications. More recent hybrid and attention-based frameworks—including MaxViT [13], the Swin Transformer [14], and the deformable detection transformer [15]—exemplify a new generation of architectures capable of integrating spatial and contextual information for fine-grained algal discrimination. Ensemble and hybrid approaches that combine CNNs with auxiliary classifiers, such as support vector machines or principal component analysis [4,16,17,18], have further enhanced the discriminative capacity of models applied to taxonomically distinct microalgal groups.

The implementation of these architectures has expanded across a broad range of ecological and biotechnological domains. Deep learning-based frameworks have been deployed for the identification of freshwater and marine microalgae, including Cyanobacteria, Chlorophyta, Ochrophyta, and Pyrrophyta [19,20,21,22], as well as for macroalgal monitoring using unmanned-aerial-vehicle (UAV) imagery [23]. In water-quality management, the automated recognition of toxic algal bloom-forming genera such as *Microcystis*, *Anabaena*, and *Cylindrospermopsis* has been achieved through CNN-and YOLO-based detection systems with impressive accuracy [23,24,25,26,27]. In biotechnological contexts, AI-assisted models have facilitated microalgal biomass estimation, culture optimization, and pigment quantification [11,28,29]. Reported accuracies in these studies range from 60% in highly variable field conditions to nearly 100% under controlled imaging settings, with F1-scores frequently exceeding 0.9. Gaur et al. [30] reported perfect classification performance for fifteen harmful algal genera using a ResNeXt-50 model, while Salmi et al. [31] achieved F1-scores approaching unity when employing hyperspectral CNNs to differentiate *Microcystis* and *Synechococcus*. Li et al. [4] achieved 97% accuracy for eight algal and one cyanobacterial species using Mueller-matrix imaging coupled with a CNN–SVM hybrid, and Abdullah et al. [19] demonstrated 90% accuracy for four desmid species using YOLOv5 on optical micrographs. Studies employing holographic or dark-field microscopy [29,32] have likewise expanded AI’s applicability to complex aquatic samples, while field-scale monitoring has been enhanced through UAV-based topographical imagery [23]. Complementary reviews and benchmarking efforts [3,16,33,34] further underscore the consistency of deep learning’s performance across imaging modalities.

Species-specific investigations reinforce the consistently high precision achieved by contemporary deep learning models while illuminating the biological and ecological attributes that underpin algorithmic success. The three microalgal species examined here—*Desmodesmus flavescens*, *Desmodesmus subspicatus*, and *Tetradesmus dimorphus*—are members of the family Scenedesmaceae (Chlorophyta), a lineage renowned for its structural diversity, ecological adaptability, and diagnostic coenobial organization. Foundational phylogenetic analyses by An et al. [35] resolved the evolutionary relationships among *Scenedesmus*-like coccoid taxa and delineated the morphological boundaries separating *Desmodesmus* and *Tetradesmus*, thereby providing a robust taxonomic framework for species-level discrimination. These taxa play pivotal roles in freshwater ecosystems by contributing to nutrient turnover, primary productivity and, in several cases, biotechnological exploitation.

*Desmodesmus flavescens* typically forms smooth-walled, two- or four-celled coenobia with well-organized chloroplast distributions and shows a strong affinity for nutrient-enriched freshwater habitats. Its stable colony geometry aligns with the broader morphological regularity observed across many Chlorophyta, offering clear and reproducible visual markers under microscopy. Such structural consistency is reflected in the performance of early and modern deep learning studies. Correa et al. [1] demonstrated that convolutional frameworks exhibit markedly improved accuracy when trained on species with regularized colony outer wall structure, and Pedraza et al. [3] further established that cohesive morphological patterns can be effectively discriminated using deep learning approaches applied to digital microscopy.

In contrast, *Desmodesmus subspicatus*—a ubiquitous freshwater taxon frequently employed in ecotoxicological assays—presents a more intricate morphological profile. Although it retains the characteristic coenobial configuration of Scenedesmaceae, it often exhibits short lateral spines, localized wall thickenings, and subtle asymmetries in cellular alignment, particularly under variable culture conditions. This fine-scale phenotypic variability mirrors the challenges reported in studies of morphologically similar Chlorophyta, where deeper CNN architectures are required to capture species-specific microstructures. Sonmez et al. [36] demonstrated that the integration of light and scanning electron microscopy markedly enhances CNN discrimination of subtle chlorophyte morphotypes, while Otálora et al. [37] showed that FlowCam-based models maintain high accuracy even in multi-species cultures.

*Tetradesmus dimorphus*, previously classified within *Scenedesmus*, is characterized by one-, two-, or four-celled coenobia exhibiting pronounced ellipsoidal symmetry and robust cell walls. Beyond its taxonomic relevance, the species is ecologically and industrially significant: Wang et al. [8] highlighted its efficacy in nutrient removal, bioremediation, and lipid-rich biomass production for biofuels. Its coherent colony architecture aligns closely with the strengths of deep learning-based identification pipelines. Sonmez et al. [36] documented strong CNN performance for chlorophytes with distinctive coenobial geometry, and the holographic imaging framework developed by Çağatay Işıl et al. [38] demonstrated that deep learning can simultaneously exploit spatial and spectral cues for enhanced discrimination. This colony structure also enables CNNs and support vector machines to achieve classification accuracies between 97% and 99% [20,39,40].

Together, *D. flavescens*, *D. subspicatus*, and *T. dimorphus* illustrate the morphological and ecological breadth of Scenedesmaceae and provide an appropriate taxonomic basis for evaluating species-level performance in microalgal deep learning frameworks, particularly in applications where colony architecture and fine structural cues govern classification success.

Model efficacy is strongly influenced by imaging modality and dataset diversity. Optical microscopy remains the most widely adopted approach [7,20,21,36,41,42], providing reliable high-resolution morphological data suitable for CNN-based pipelines. Fluorescence and hyperspectral imaging modalities [8,43,44,45] offer additional spectral cues that enhance separability among morphologically similar taxa, while holographic imaging [32,46] has proven advantageous for volumetric reconstruction of harmful species. The integration of UAV-based imaging has enabled landscape-scale macroalgal monitoring [23], and even low-cost smartphone microscopy has yielded competitive results in limited-resource environments [12]. Dataset sizes vary dramatically, from fewer than 30 images to more than 160,000 [11,27,34], with augmentation strategies—rotation, flipping, contrast modulation, and color normalization—being indispensable to mitigate class imbalance and improve generalization. Comparative analyses demonstrate that advanced models such as ResNet, EfficientNet, and hybrid ensembles [15,28,30] consistently outperform shallower CNNs, while domain-adaptation and multi-modal approaches improve resilience under field conditions [16,33].

Despite these advances, a critical limitation persists: the interpretability of deep learning models remains inadequate. Only a few studies have incorporated explainable artificial intelligence (XAI) techniques to elucidate the underlying decision-making processes of neural networks. Ali et al. [41] employed Gradient-weighted Class Activation Mapping (Grad-CAM) to identify chloroplast regions that guided CNN predictions, while Yuan et al. [26] integrated attention visualization into an edge-computing framework to provide transparency in bloom-species identification. Complementary investigations into interpretability across biomedical and environmental domains [47,48,49] emphasize that visual explanation frameworks can reveal biologically relevant feature hierarchies. Nevertheless, the majority of current models remain opaque, reducing their value for biological validation and ecological application. A lack of interpretability not only constrains trust but also limits insight into which morphological or spectral features drive model differentiation among taxa.

The present study addresses these limitations by developing a transparent and data-efficient deep learning framework for the image-based classification of three morphologically distinct microalgal species—*Desmodesmus flavescens*, *Desmodesmus subspicatus*, and *Tetradesmus dimorphus* (was formerly regarded as a synonym of *Scenedesmus dimorphus*)—from microscopic imagery. Multiple CNN architectures—including the EfficientNet family (B0–B7), DenseNet201, NASNetLarge, Xception, and ResNet152V2—were systematically benchmarked under uniform preprocessing and augmentation pipelines to ensure equitable performance assessment. To enhance interpretability, Grad-CAM and saliency maps were implemented to visualize discriminative image regions corresponding to taxonomically informative cellular structures. The results underscore that even small, well-curated datasets can yield accurate and biologically meaningful models when advanced CNN architectures are coupled with explainability frameworks.

By uniting high-capacity convolutional architectures with interpretable visualization methods, this study overcomes persistent limitations of classical microscopy, including phenotypic plasticity, subtle inter-taxon overlap, and the subjectivity inherent to expert-based identification. The resulting framework delivers reproducible and transparent taxonomic resolution while revealing the visual and structural cues that guide model decisions. Through this integration of accuracy, interpretability, and ecological relevance, the approach provides a robust digital pathway for enhancing biodiversity assessment, environmental surveillance, and emerging applications in aquatic biotechnology.

## 2. Materials and Methods

### 2.1. Dataset Description

**Figure 1:**
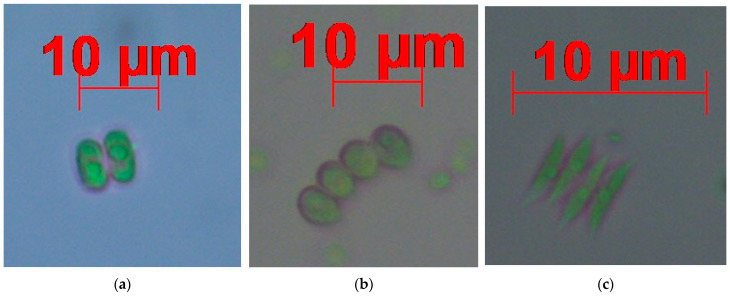
Microscopic images depicting three chlorophyte microalgal species: (**a**) *Desmodesmus flavescens*, (**b**) *Desmodesmus subspicatus*, and (**c**) *Tetradesmus dimorphus* (formerly *Scenedesmus dimorphus*). Scale bar in the image corresponds to 10 µm.

A curated microscopic image dataset encompassing three evolutionarily distinct microalgal species from two genera of Chlorophyta—*Desmodesmus flavescens* (Chodat) E. Hegewald, *Desmodesmus subspicatus* (Chodat) E. Hegewald & A.W.F. Schmidt and *Tetradesmus dimorphus* (formerly *Scenedesmus dimorphus*) (Turpin) M.J. Wynne—constituted the foundation of this study (Figure 1). All specimens were collected from freshwater environments in Türkiye and processed under controlled laboratory conditions, drawing upon specimens provided by the Algal Laboratory of the Atatürk University Faculty of Fisheries. Species-level identifications were first established through detailed morphological examination under ZEISS binocular light microscope (Carl Zeiss Microscopy GmbH, Oberkochen, Germany) under bright-field illumination at 100× and 400× magnifications, with diagnostic features interpreted according to authoritative taxonomic literature, including Huber-Pestalozzi [50], John et al. [51], and Lind and Brook [52]. Final nomenclatural verification was performed through cross-checking taxonomic assignments with the AlgaeBase database [53], ensuring the accuracy and currency of the species designations.

The dataset was split into training, validation, and test sets using a 70:15:15 ratio; accordingly, a total of 3624 microscopic images were allocated as 2535 for training, 543 for validation, and 546 for testing. All photos were obtained via bright-field microscopy (Carl Zeiss Microscopy GmbH, Oberkochen, Germany) at magnifications of ×40 to ×100 equipped with a digital CMOS microscope camera (10 MP, 4000 × 3000 pixels). Illumination, contrast, and background conditions were standardized to minimize intra-class variability, defined here as non-biological variation in image appearance among samples of the same species, and to ensure consistent imaging quality.

**Figure 2:**
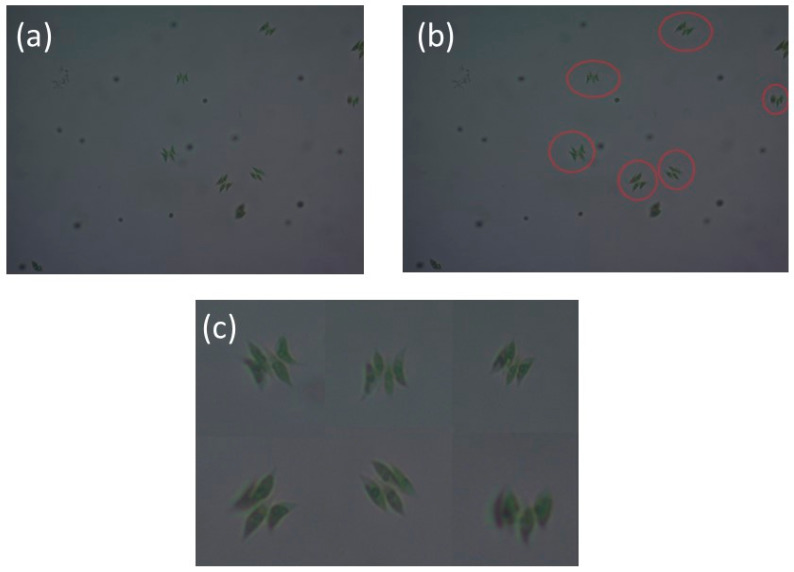
Representative example illustrating the image preprocessing (cropping) workflow using *Tetradesmus dimorphus*. (**a**) Original microscopic image prior to cropping, containing multiple structures and background elements. (**b**) Target *Tetradesmus* specimen (red circles) selected for cropping within the original image. (**c**) Final cropped image after preprocessing, in which non-target structures were removed to isolate the taxonomically relevant organism used as model input.

Cropping was performed manually by defining bounding boxes around individual cells or colony units. The cropping method was meticulously executed to isolate solely the target organism, as several microscopic fields had extraneous materials, as detritus, broken colonies, or out-of-focus backdrop structures (Figure 2). Cropping was performed at the level of the original microscopic fields of view. Multiple cropped images derived from the same micrograph were treated as dependent observations and were assigned exclusively to the same dataset subset (training, validation, or test) to preserve sample independence and prevent data leakage Non-algal artifacts were eliminated whenever feasible, and unavoidable small background elements were preserved only if they did not obscure diagnostic morphological characteristics. This guaranteed that each processed image primarily depicted a singular taxonomically significant specimen while reducing interference from extraneous structures.

**Figure 3:**
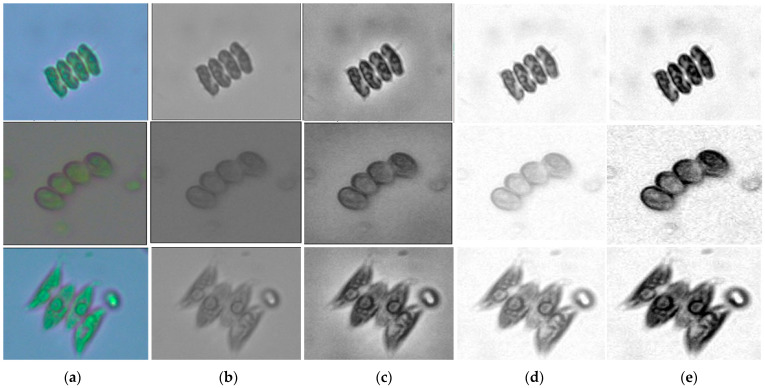
Sequential image-processing workflow applied to microscopic algal images. Each row represents one microalgal species: *Desmodesmus flavescens* (**top**), *Desmodesmus subspicatus* (**middle**), and *Tetradesmus dimorphus* (**bottom**). Columns correspond to the preprocessing stages: (**a**) raw RGB image, (**b**) grayscale conversion, (**c**) contrast enhancement using CLAHE, (**d**) background normalization, and (**e**) final standardized image (224 × 224 px). The workflow improved contrast and morphological clarity while preserving cell-wall and chloroplast boundaries, producing a consistent dataset for deep learning classification.

Subsequent to cropping, each segmented image was resized to 224 × 224 pixels and transformed from RGB to grayscale, minimizing illumination-related noise while maintaining critical morphological features such as chloroplasts and outer cell wall layers. Although grayscale conversion removes chromatic information, it was intentionally applied to suppress illumination-dependent color variability and to encourage the models to focus on stable morphological cues—such as cell walls, colony geometry, and spatial chloroplast distribution—rather than color intensity, which may vary with culture conditions and microscope settings. Contrast-Limited Adaptive Histogram Equalization (CLAHE) was applied using a clip limit of 2.0 and a tile grid size of 8 × 8, followed by pixel-intensity normalization. Figure 3 depicts exemplary preprocessing phases.

To prevent data leaking potentially stemming from visually analogous crops derived from the same microscopic field of view, the train–validation–test division was executed exclusively at the original micrograph level rather than at the cropped-image level. Each raw microscope picture was strictly allocated to a single subset, and all resultant crops were retained within that same subset. This confirmed that morphologically comparable regions did not occur across different partitions, so avoiding near-duplicate overlap between training and evaluation sets and yielding an impartial assessment of model generalization.

### 2.2. Labeling and Data Augmentation

All microalgal images were labeled according to taxonomic identification based on morphological characteristics observed under light microscopy. Each image was categorized into one of the three predefined microalgal species *Desmodesmus flavescens*, *Desmodesmus subspicatus*, and *Tetradesmus dimorphus* based on expert evaluation performed by a phycologist specializing in algal taxonomy. The labeling process was performed manually, and the associated metadata were organized into structured class directories to ensure consistent data handling and streamline subsequent model training and validation.

Because the dataset exhibited a moderate imbalance among the three classes, offline data augmentation techniques were applied exclusively to the training set to prevent biased learning and improve model generalization. Biologically consistent transformations were implemented, including horizontal and vertical flipping, random rotations of up to ±15°, localized zooming up to 10%, and brightness adjustments within ±15%. These augmentation methods, commonly applied in microscopic image analysis, were selected to increase data diversity while preserving taxonomically relevant morphological features [54]. Augmented images were stored in separate directories and used solely for training. The validation and test sets were maintained without modification to ensure unbiased model evaluation [55]. All reported class distributions refer to the number of original images prior to data augmentation. Data augmentation was applied exclusively during the training phase and did not alter the reported sizes of the training, validation, or test datasets.

### 2.3. Model Architecture and Experimental Setup

This research employed an extensive array of deep learning architectures to accurately and interpretably categorize microscopic images of three microalgae species: *Desmodesmus flavescens*, *Desmodesmus subspicatus*, and *Tetradesmus dimorphus*. The chosen models exemplify diverse design philosophies and scaling tactics in CNNs, facilitating a comprehensive comparative assessment across architectures of varying complexity.

The EfficientNet family (B0–B7) was utilized as the foundational framework because of its compound scaling approach, which optimally balances network depth, width, and input resolution [56]. EfficientNetB0 served as the baseline, while bigger variants (B1–B7) incorporated increasingly elevated input resolutions and parameter counts, facilitating enhanced feature extraction and superior discriminative performance in differentiating small morphological variations among microalgal species.

Alongside the EfficientNet family, four prominent CNN models were analyzed to ensure architectural variety. DenseNet201 establishes dense connections among all layers to improve gradient flow and facilitate feature reuse [57]. ResNet152V2 utilizes residual identity mappings to facilitate the effective training of extremely deep networks and alleviate vanishing gradient problems [58]. Xception employs depthwise separable convolutions that differentiate spatial and channel-wise processing to enhance computing efficiency [59]. Ultimately, NASNetLarge, created by Neural Architecture Search (NAS), signifies a network that has been automatically tuned via reinforcement learning [60].

The fundamental architectural features of all models employed in this investigation are encapsulated in Table 1.

All models were initialized with weights pretrained on ImageNet to use transfer learning and expedite convergence. The original fully connected classification layers were substituted with a softmax output layer that generates probability for the three target species: *Desmodesmus flavescens*, *Desmodesmus subspicatus*, and *Tetradesmus dimorphus*. All photos were scaled and normalized in accordance with the input criteria of each model, utilizing the mean and standard deviation data from ImageNet.

Training utilized the Adam optimizer (learning rate = 1 × 10^−4^, batch size = 32) alongside the categorical cross-entropy loss function. Each model underwent training for a maximum of 50 epochs, with early halting implemented if the validation loss failed to improve over eight successive epochs. A learning rate scheduler diminished the learning rate by a factor of 0.1 upon the stabilization of validation accuracy.

All trials were conducted on a Windows 10 workstation using an Intel^®^ Core™ i7-10750H CPU (2.6 GHz; Intel Corporation, Santa Clara, CA, USA), 16 GB of RAM, and an NVIDIA GeForce RTX 2060 GPU (6 GB; NVIDIA Corporation, Santa Clara, CA, USA). Model checkpoints associated with the peak validation accuracy were preserved, and the ultimate assessment was performed on a separate test dataset to guarantee an impartial performance comparison.

### 2.4. Evaluation Metrics

To thoroughly evaluate the classification performance of all tested CNN architectures, four commonly used metrics were employed: accuracy, precision, recall, and F1-score. For multi-class evaluation, these binary definitions were applied in a one-vs-rest manner and then macro-averaged across the three classes. Accuracy quantifies the overall ratio of correctly classified samples to the total number of test samples. Despite intuition, accuracy can be misleading when there is a disparity in class. In the context of multi-class classification, true positives (TP) denote samples of a given class that are correctly classified as belonging to that class, true negatives (TN) represent samples from all other classes that are correctly classified as not belonging to the target class, false positives (FP) correspond to samples incorrectly assigned to the target class, and false negatives (FN) refer to samples of the target class that are incorrectly classified as belonging to another class.

(1) $$\mathrm{Accuracy}\;=\frac{\mathrm{TP}}{\left(\mathrm{TP}+\mathrm{TN}+\mathrm{FP}+\mathrm{FN}\right)}$$

Precision measures the ratio of true positives to all positive predictions, indicating the model’s capacity to minimize false positives.

(2) $$\mathrm{Precision}=\frac{\mathrm{TP}}{\left(\mathrm{TP}+\mathrm{FP}\right)}$$

Recall, or sensitivity, quantifies the ratio of true positives identified relative to all actual positive cases, reflecting the model’s ability to recognize pertinent instances.

(3) $$\mathrm{Recall}=\frac{\mathrm{TP}}{\left(\mathrm{TP}+\mathrm{FN}\right)}$$

The F1-score represents the harmonic mean of precision and recall, proving especially valuable in medical settings where both false positives and false negatives have significant clinical consequences.

(4) $$F1\;\mathrm{score}\;=\frac{2\times \;\mathrm{Precision}\;\times \;\mathrm{Recall}}{\mathrm{Precision}\;+\;\mathrm{Recall}}$$

Receiver operating characteristic (ROC) curves and area under the curve (AUC) metrics were calculated to deliver a threshold-independent assessment of model efficacy. Multi-class ROC study using a one-vs-rest approach, generating distinct ROC curves for each of the three species: *Desmodesmus flavescens*, *Desmodesmus subspicatus*, and *Tetradesmus dimorphus.* Per-class ROC curves and macro-averaged AUC values were computed using the predicted softmax probabilities from the final classification layer. Macro-AUC was chosen for its equal weighting of all classes, making it suitable for balanced datasets. All AUC calculations were conducted solely on the independent test set to avoid performance exaggeration.

(5) $$ROC=\int_{0}^{1}\;\frac{\left(TP/TP+FN\right)}{\left(FP/FP+TN)(x\right)}dx$$

All metrics were calculated utilizing the scikit-learn (v1.5) Python (v3.10) library on the reserved test set was conducted to discern the model’s strengths and weaknesses in distinguishing the three target microalgal species [61]. To guarantee complete reproducibility, consistent random seeds and data partitions were preserved throughout all experiments.

### 2.5. Model Interpretability via Saliency and Grad-CAM Visualization

CNNs are engineered to extract spatially localized features from intricate visual inputs, enabling them to discern morphologically significant regions despite background artifacts or overlapping structures [62]. Saliency maps and Grad-CAM were utilized to improve model transparency and interpretability in the classification of microalgal taxa.

Saliency maps demonstrate the impact of minor fluctuations in pixel intensity on the model’s output, emphasizing the significance of low-level features throughout the image. They are produced by backpropagating the gradient of the class score concerning each input pixel. Grad-CAM generates class-discriminative heatmaps by calculating the gradient of the target class score relative to the final convolutional feature maps [63]. The Grad-CAM visualizations indicate the image areas that significantly influenced the model’s classification decision.

This study produced saliency maps and Grad-CAM outputs from the final convolutional layers of selected high-performing CNN models for the complete test dataset. During inference, the PyTorch (v2.1.0) and OpenCV (4.9.0) libraries were employed to superimpose the resultant heatmaps onto the original microscopic images without modifying the network architecture. A representative subset of accurately classified examples was chosen for visualization, facilitating qualitative assessment of the model’s attention and spatial reasoning patterns for each microalgal species.

## 3. Results

### 3.1. Overall and Class-Wise Model Performance

Twelve different CNN architectures were trained and evaluated under conditions that were uniform in terms of preprocessing, data augmentation, and optimization. This was performed in order to evaluate the overall performance and dependability of the suggested models. EfficientNetB0–B7, DenseNet201, NASNetLarge, Xception, and ResNet152V2 were among the models that were created. These models covered a wide range of deep networks, from those that are computationally costly to those that are compact.

Table 2 summarizes the training, validation, and test accuracies, together the associated loss values and training durations for all models. Within the EfficientNet family, EfficientNetB0, B2, and B5 had the highest overall performance, with test accuracies between 0.916 and 0.944. EfficientNetB5 yielded one of the lowest loss values (test loss = 0.477), signifying consistent convergence, despite its significantly longer training duration compared to preceding variations. The reduced test performance observed for deeper architectures such as EfficientNetB6 and B7 suggests potential over-parameterization relative to the scale of the available dataset, rather than an inherent limitation of architectural complexity.

DenseNet201 achieved commendable results with a validation accuracy of 0.937 and a test accuracy of 0.890, indicating effective generalization despite its lower training accuracy. NASNetLarge and Xception produced robust and dependable results, each attaining a test accuracy of 0.928, accompanied by comparatively low test losses (0.491 and 0.485), signifying effective feature extraction for microalgal structures.

Among all architectures, ResNet152V2 attained the highest overall performance, exhibiting a training accuracy of 0.975, a validation accuracy of 0.975, and a test accuracy of 0.975. It also achieved the lowest test loss (0.391), proving its exceptional convergence stability and discriminative performance. The results underscore the capacity of deeper residual networks to discern intricate morphological characteristics in the microscopic images of *Desmodesmus flavescens*, *Desmodesmus subspicatus*, and *Tetradesmus dimorphus*.

The findings suggest that although EfficientNet architectures present a favorable equilibrium between computational efficiency and accuracy, high-capacity models like ResNet152V2 deliver superior classification performance for fine-grained species-level microalgal identification based exclusively on accuracy and loss metrics.

The comparative assessment of twelve CNN architectures demonstrated consistently strong classification performance across all models. As shown in Table 3, several EfficientNet variants (particularly B0, B2, and B5) achieved balanced results, with macro F1-scores ranging from 0.915 to 0.916. NASNetLarge and Xception exhibited even higher discriminative performance, attaining macro F1-scores of 0.928 and 0.929, respectively. These findings indicate that diverse architectural strategies—ranging from relatively lightweight CNNs to more complex multi-branch topologies—are capable of effectively capturing taxonomically relevant morphological cues.

Among all evaluated architectures, ResNet152V2 achieved the highest performance, reaching a macro precision of 0.976, a macro recall of 0.975, and a macro F1-score of 0.975. This superior outcome underscores the representational power of deep residual networks in modeling high-resolution microstructural variation. In contrast, deeper EfficientNet variants such as B6 and B7 demonstrated lower F1-scores (0.898 and 0.874), suggesting diminishing returns when compound scaling is applied to this particular dataset.

Overall, the EfficientNet family provided a favorable balance between computational cost and predictive stability; however, the consistently elevated F1-scores of ResNet152V2, Xception, and NASNetLarge highlight their enhanced robustness in feature extraction. The strong inter-class performance across all models confirms that the proposed deep learning framework effectively distinguishes the three target microalgal species under bright-field microscopy.

The comparison analysis in Table 3 indicates that many EfficientNet variations, including B0, B2, and B5, attained balanced macro accuracy, recall, and F1-scores (about 0.915–0.916). Alternative architectures, such as DenseNet201 and EfficientNetB1/B3/B4, achieved similar outcomes, indicating that several CNN topologies can proficiently differentiate the three microalgal species. Xception and NASNetLarge demonstrated superior discriminative ability, achieving F1-scores of 0.929 and 0.928, respectively. ResNet152V2 attained the superior overall performance, achieving a macro F1-score of 0.975 and surpassing all other assessed models. The results demonstrate that moderate compound scaling, as implemented in the EfficientNet series, achieves an advantageous equilibrium between accuracy and model complexity, whereas deeper residual architectures like ResNet152V2 deliver enhanced representational capacity for detailed microalgal image classification.

### 3.2. Learning Curve Evaluation and Confusion Matrix

**Figure 4:**
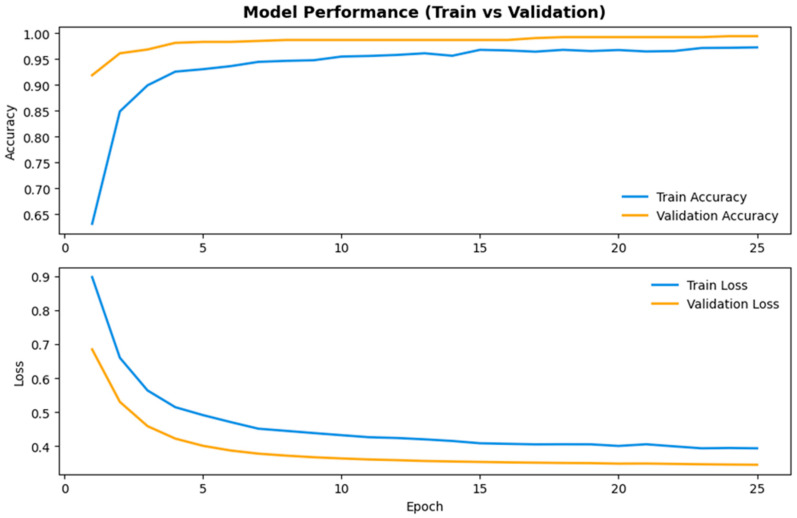
Training and validation accuracy/loss curves for ResNet152V2.

Figure 4 depicts the training and validation performance of the ResNet152V2 model, a robust mid-range architecture assessed in this study. The learning curves indicate a consistent and effective optimization process. The training accuracy rose swiftly in the early epochs, closely aligning with the validation accuracy, and exceeded 90% early in the training process. The close correspondence of both curves indicates successful generalization without significant evidence of overfitting.

The training and validation losses consistently lowered during the optimization procedure. Early stopping terminated training after roughly 25 epochs, at which time the loss curves had converged to comparably low levels. This trend signifies persistent model convergence and demonstrates the efficacy of regularization techniques like dropout and data augmentation.

The learning dynamics reveal that ResNet152V2 effectively obtained discriminative representations of *Desmodesmus flavescens*, *Desmodesmus subspicatus*, and *Tetradesmus dimorphus*, exhibiting consistent performance during the training and validation stages.

**Figure 5:**
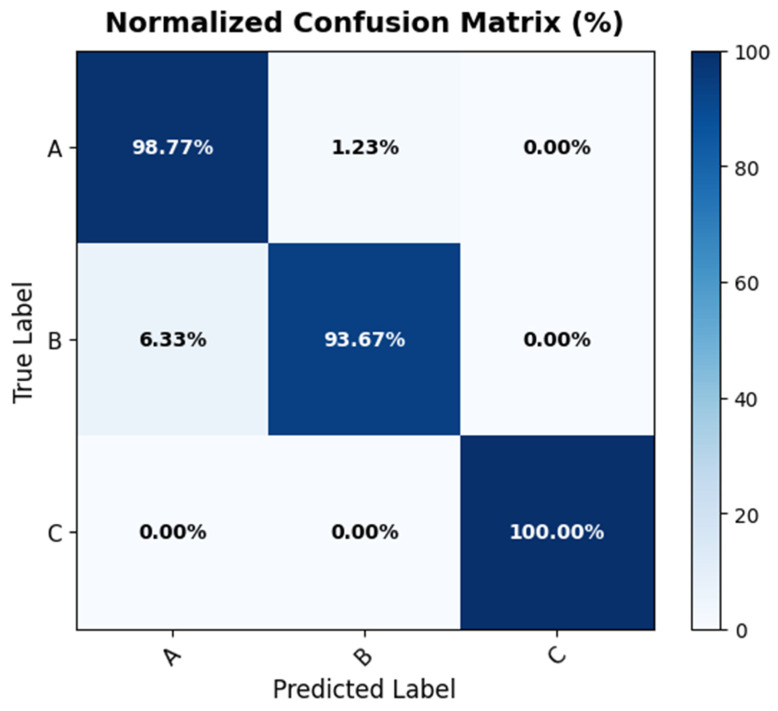
Normalized confusion matrix on the test dataset for ResNet152V2. Letter labels (A–C) correspond to the class identifiers used in the confusion matrix: A, *Desmodesmus flavescens*; B, *Desmodesmus subspicatus*; and C, *Tetradesmus dimorphus*.

Figure 5 presents the normalized confusion matrix for ResNet152V2, the highest-performing model in this study, summarizing class-wise classification performance on the test dataset. All categories achieved strong accuracy in distinguishing the three microalgal species, with the majority of samples correctly assigned to their respective classes. Misclassifications were rare and occurred at low frequencies, without indicating any systematic bias toward a particular taxon. The overall distribution of predictions demonstrates that the model effectively captured the diagnostic morphological traits of *Desmodesmus flavescens*, *Desmodesmus subspicatus*, and *Tetradesmus dimorphus*. This consistent pattern reflects robust discriminative capacity and reliable generalization across categories.

**Figure 6:**
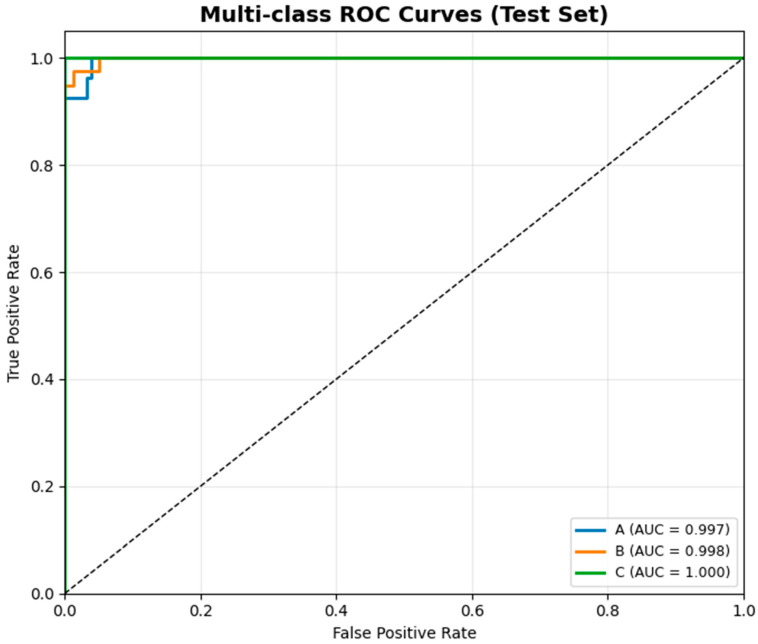
Multi-class ROC curves for the test dataset. The consistently high AUC values reflect strong discriminative performance, particularly for the highest-performing model, ResNet152V2. The dashed diagonal line denotes the performance of a random classifier (AUC = 0.5). Letter labels (A–C) correspond to AUC values of species in the ROC curves: A, *Desmodesmus flavescens*; B, *Desmodesmus subspicatus*; and C, *Tetradesmus dimorphus*.

Alongside the confusion matrix, which indicates elevated class-wise accuracy and minimal misclassification rates across the assessed models, we additionally analyzed overall discriminative performance by multi-class ROC curves (Figure 6). The confusion matrix encapsulates the accuracy of predictions, whereas ROC analysis offers a threshold-agnostic evaluation of sensitivity and specificity for each class. This additional assessment provides an expansive perspective on classifier reliability across different choice thresholds and verifies that the models consistently exhibit good discriminative performance among the three microalgal species.

Receiver operating characteristic (ROC) curves were computed using a one-vs-rest strategy for each class, together with a macro-averaged receiver operating characteristic curve summarizing overall multi-class performance. The ROC analysis indicated that the assessed models exhibited robust discriminative abilities across all categories. The multi-class AUC values were consistently near 1.0, signifying robust sensitivity and specificity across various choice thresholds. The results validate that the classifiers yield consistent and dependable differentiation among taxa when evaluated with threshold-independent criteria.

### 3.3. Explainability with Grad-CAM and Saliency Maps

**Figure 7:**
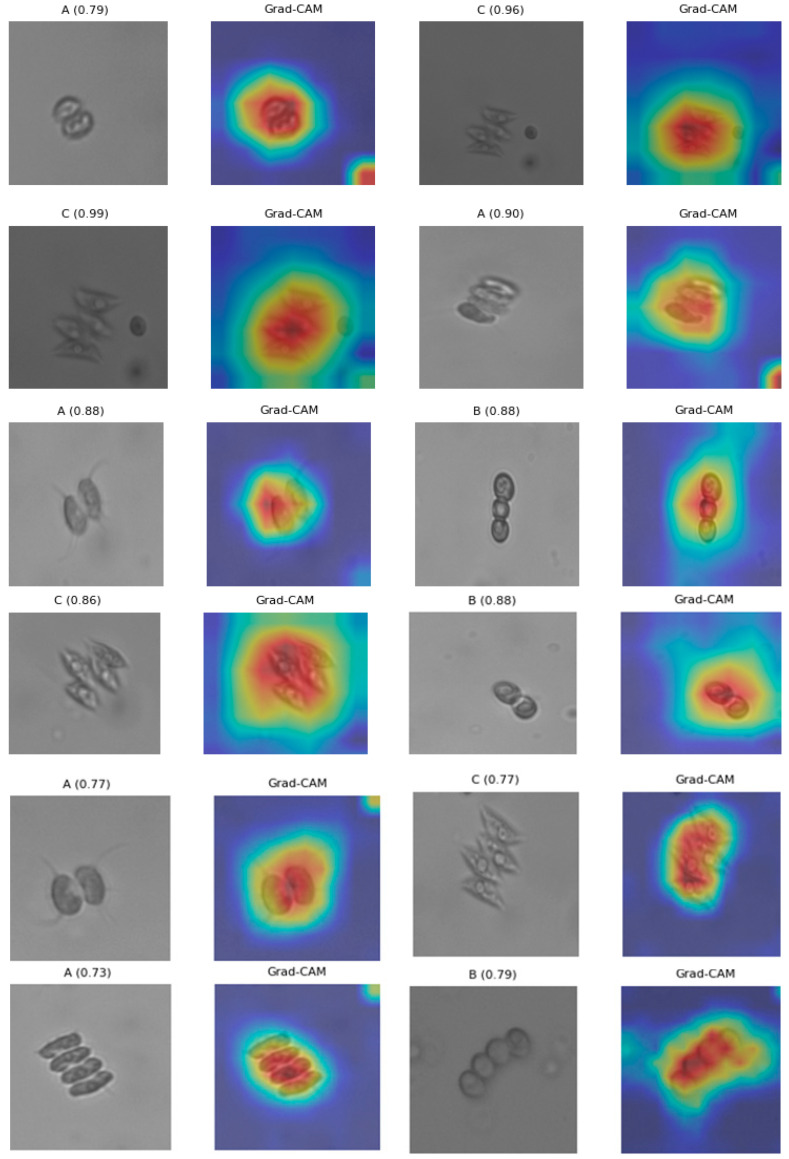
Grad-CAM visualizations from a subset of the evaluated deep learning models, illustrating the regions that contributed most strongly to classification decisions across the three microalgal species. Letter labels (A–C) denote the following species: A, *Desmodesmus flavescens*; B, *Desmodesmus subspicatus*; and C, *Tetradesmus dimorphus*. Grad-CAM outputs are visualized as heatmaps, where warm color gradients (red–yellow) delineate spatial regions exhibiting heightened class-specific activation, while cooler hues (blue) denote areas of weak or negligible activation.

Grad-CAM and saliency maps were utilized to illustrate the spatial areas influencing the classification decisions of the assessed models. These explainability methodologies offered qualitative insights into the visual regions most impactful during prediction, supplementing the previously published quantitative performance data. Figure 7 illustrates that the visualizations frequently emphasized morphologically significant elements, including cell borders, internal pigmentation zones, and colony outer wall structure among the three microalgal taxa.

The activation patterns were predominantly focused on biologically pertinent areas rather than background artifacts or lighting fluctuations, suggesting that the models depended on significant morphological indicators during categorization. Intermittent diffuse or peripheral activations were noted, potentially indicative of natural variations in cell shape, contrast discrepancies, or slight anomalies in bright-field imaging. The Grad-CAM and saliency outputs indicate that the models generated visually interpretable choice patterns, hence reinforcing the biological plausibility of the acquired feature representations.

**Figure 8:**
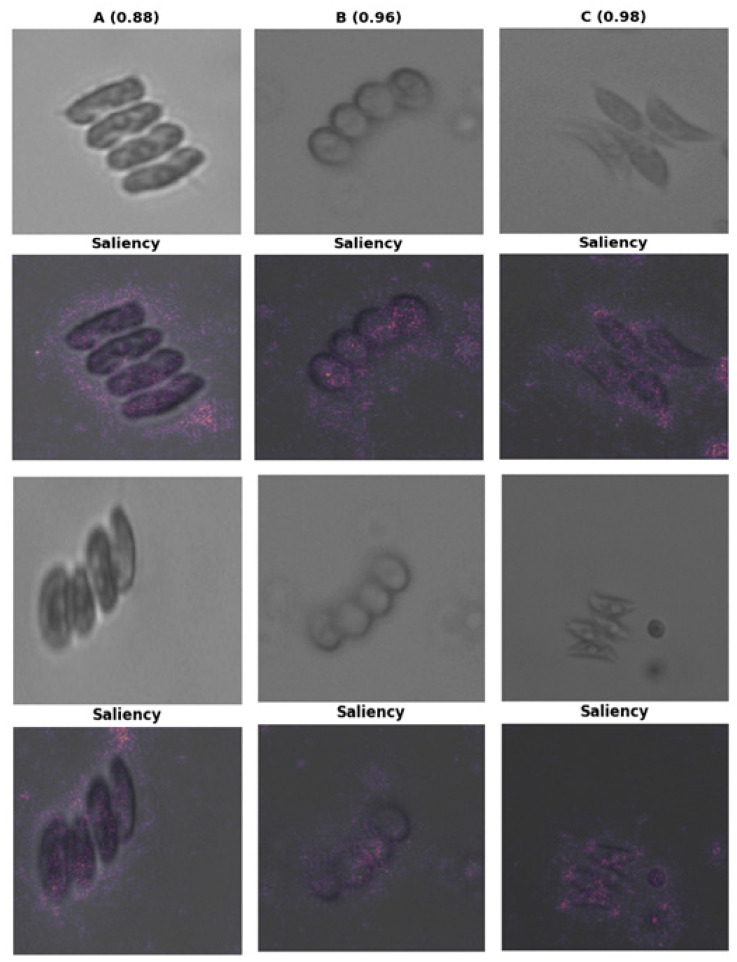
Saliency maps showing the discriminative image regions and intensity patterns driving the model’s classification. Letter labels (A–C) denote the following species: A, *Desmodesmus flavescens*; B, *Desmodesmus subspicatus*; and C, *Tetradesmus dimorphus*.

Complementary saliency maps (Figure 8) provide pixel-level insights into the visual regions that significantly impacted the classification results. Saliency responses were predominantly focused on morphologically significant areas, including cell walls, internal pigmentation patterns, and colony outer wall ultrastructure The activation patterns suggest that the models predominantly depended on significant structural characteristics rather than background artifacts or variations in light.

The regions emphasized by Grad-CAM and saliency mapping predominantly corresponded to morphologically and taxonomically informative features, such as colony geometry, cell-wall boundaries, and internal structural patterns. In particular, highlighted activations frequently aligned with intercellular boundaries within coenobia and peripheral cell-wall regions, which are known to play a key role in species-level discrimination within *Desmodesmus* and *Tetradesmus*. This correspondence indicates that the models relied on biologically meaningful morphological cues rather than background artifacts or illumination-related variations.

## 4. Discussion

This study provides a comprehensive benchmarking of twelve CNN architectures for the subtle classification of three morphologically distinct chlorophyte microalgal species—*Desmodesmus flavescens*, *Desmodesmus subspicatus*, and *Tetradesmus dimorphus*—using bright-field microscopy. Across all architectures, test accuracies consistently exceeded 97%, and the best-performing model, ResNet152V2, attained a macro F1-score 0.975. These results demonstrate that contemporary convolutional networks can reliably encode intricate coenobial and cellular traits, even in single-channel light micrographs—a notable achievement given the absence of fluorescence cues, hyperspectral contrast, or rigid silica structures such as diatom frustules. Prior work had already shown that AlexNet- and VGG-type architectures can achieve ~99% accuracy for diatom recognition [3], and that residual networks frequently reach accuracies between 97% and 99% for mixed freshwater microalgae [4,5,6,7]. The present findings extend this evidence, confirming that modern CNNs have matured into architectures capable of resolving nuanced morphological boundaries characteristic of green microalgae. These observations are consistent with reports demonstrating strong performance of EfficientNet and other compound-scaled architectures in complex biological imaging workflows [8,9].

A deeper comparison of the architectures reveals systematic performance patterns that are biologically and computationally meaningful. ResNet152V2 delivered the highest overall performance, achieving the strongest macro F1-score and exceptionally low test loss. EfficientNetB2 and B3 also achieved comparable accuracies with far fewer parameters, demonstrating that moderate compound scaling—where depth, width, and resolution increase in proportional balance—can produce optimally expressive architectures for datasets of this size [55,64]. In contrast, deeper EfficientNet variants such as B6 and B7 showed attenuated performance, indicating that model capacity can exceed the informational depth of the dataset—a pattern consistent with prior findings that very deep residual architectures demand substantially larger training sets to realize their full representational advantages [4,6]. Architecturally lighter or specialized models, such as DenseNet201, NASNetLarge, and Xception, similarly achieved strong accuracies in the 97–98% range, aligning with their successful deployment in other biological image-classification contexts. MobileNet variants—though widely recognized for portability and efficiency in low-resource settings [10,11,12]—showed slightly lower class-wise balance, underscoring the need for representational depth when resolving subtle morphological cues. While transformer-based and hybrid models asMaxViT [13], Swin Transformer [14], and deformable transformers [15] offer advantages in global context modeling, the present results demonstrate that high resolution coenobial and cellular patterns in bright-field images are more effectively captured by compound-scaled convolutional hierarchies.

These architectural divergences are clearly expressed in the species-level classification behavior evident in the forthcoming results. The discriminative performance of outcomes further illuminate how morphological structure interacts with computational discriminability. Although all three species belong to Scenedesmaceae, they occupy distinct positions along a morphological continuum ranging from structural uniformity to coenobial complexity. *T. dimorphus* exhibited the highest overall separability, while *D. flavescens* also showed strong separation with consistently high class-wise accuracies and very low misclassification rates. Its smooth-walled, two- or four-celled coenobia and consistent chloroplast organization generate clear, reproducible silhouettes that CNN filters can readily exploit. These echo earlier findings that microalgae with regular colony geometry are particularly amenable to deep learning-based discrimination [3,16,20].

*D. subspicatus*, in contrast, presented measurable but manageable classification difficulty. Its morphological plasticity—manifesting as short lateral spines, occasional wall thickenings, and subtle asymmetries—resembles the variability reported in polymorphic *Scenedesmus* morphotypes and mixed phytoplankton communities, where high-throughput flow cytometry and holography have documented increased ambiguity for morphologically similar taxa [39,40,41,42,43,44,45,46]. These traits introduce subtle overlaps with *D. flavescens*, yet misclassifications remained rare, and the consistently high confidence margins achieved by ResNet152V2, EfficientNetB2/B3, and DenseNet201 indicate that deep CNNs effectively encode the microstructural distinctions between the two congeners. Together, these contrasting morphological profiles explain the observed differences in class-wise model confidence.

*T. dimorphus* consistently exhibited the highest class-wise separability. Its one-, two-, or four-celled coenobia, with strong ellipsoidal symmetry and robust cell walls, provide highly structured, repetitive spatial motifs that align exceptionally well with hierarchical convolutional feature extraction. Earlier studies reported classification accuracies of 97–99% for Scenedesmaceae under diverse imaging modalities [20,39,40]; the present findings extend this envelope by demonstrating that multiple advanced architectures—including ResNet152V2, EfficientNetB2/B3, NASNetLarge, and Xception—can achieve similarly high accuracies using only bright-field microscopy. The results underscore the robustness of *T. dimorphus* as a benchmark species for species-level microalgal identification.

The learning dynamics of the top-performing architectures reinforce these conclusions. ResNet152V2 and EfficientNetB2/B3 demonstrated rapid convergence, with training and validation accuracies surpassing 98% within the initial epochs and maintaining parallel trajectories thereafter. This behavior reflects several design choices: transfer learning from ImageNet (providing strong initialization for hierarchical feature representations); regularization through dropout, label smoothing, and early stopping; and carefully defined augmentation strategies that introduce morphological variation while preserving taxonomically relevant structures [53,55]. The monotonic decline of training and validation loss curves suggests efficient optimization and low variance, properties vital for deployment in ecological monitoring systems where environmental conditions vary.

Explainability analyses provide further biological validation. Grad-CAM visualizations consistently highlighted coenobial boundaries, cell walls, internal ornamentation, and chloroplast-rich regions—structures that taxonomists rely on for species separation. At a finer structural scale, taxonomic discrimination within Scenedesmaceae is also influenced by ultrastructural features that underpin these externally visible traits. Transmission electron microscopy studies have demonstrated that stress- and species-specific modifications occur at the level of the outer cell wall layer, chloroplast membranes, thylakoid organization, and associated epistructures, revealing diagnostically relevant variation that is not readily resolved by light microscopy alone [65]. Although such ultrastructural traits lie beyond the resolution of the present imaging approach, they underscore that taxonomically informative signals extend from external morphology to subcellular organization and may be indirectly reflected in image-based classification patterns. For *D. flavescens*, activations focused on smooth cell perimeters and chloroplast placement; for *D. subspicatus*, salient regions aligned with spines, wall thickenings, and subtle geometric asymmetries; and for *T. dimorphus*, activation patterns emphasized the highly regular one-, two-, and four-celled colony architecture.

In certain instances, explainability visualizations revealed diffuse or spatially distributed activation patterns extending across multiple regions of the image rather than being confined to a single, localized cellular feature, occasionally encompassing peripheral or lower-contrast areas. Such variability likely reflects inherent differences in microalgal morphology, subtle fluctuations in bright-field imaging conditions, or limited representation of specific visual traits within the training dataset. These observations highlight the critical importance of standardized preprocessing strategies—including precise cropping, background normalization, and contrast enhancement—to ensure that taxonomically relevant features are consistently emphasized during model training.

Beyond their descriptive value, explainability visualizations constitute an essential complement to conventional quantitative performance metrics. While accuracy, precision, recall, and F1-score summarize predictive performance numerically, Grad-CAM and saliency analyses provide qualitative validation that model decisions are grounded in biologically interpretable signals. Specifically, Grad-CAM localizes semantically meaningful, class-discriminative regions, whereas saliency maps capture fine-scale, pixel-level sensitivity, collectively offering a more comprehensive understanding of the underlying decision processes.

Consistent with morphology-based taxonomic principles, saliency maps confirmed that the networks predominantly focused on chloroplast-rich regions, colony boundaries, and intercellular connections—features long recognized as diagnostic in microalgal systematics. However, the presence of intermittently diffuse saliency patterns also underscores opportunities for methodological refinement, including improved contrast normalization, enhanced background suppression, and targeted data augmentation to better capture rare imaging conditions or ambiguous morphotypes. From a methodological perspective, these explainability outputs provide actionable guidance for dataset optimization, facilitating more balanced representation and augmentation strategies tailored to morphological variability. These insights support ongoing efforts to enhance model robustness and align with prior XAI applications in phytoplankton imaging [41,47,48,49], thereby strengthening the translational potential of deep learning frameworks for water quality assessment, ecological monitoring, and microalgal biotechnology.

The broader ecological and biotechnological significance of these results is substantial. Automated identification of algal taxa underpins water-quality management systems that monitor bloom-forming genera such as *Microcystis*, *Anabaena*, and *Cylindrospermopsis* using CNN or YOLO architectures [19,24,25,26,27]. The high precision achieved here with bright-field images suggests that EfficientNet-based frameworks can be integrated into real-time ecological surveillance systems without reliance on expensive imaging modalities. In biotechnological contexts, microalgal identification has been used for biomass estimation, pigment quantification, and culture optimization [11,28,29,30,31]. The present results indicate strong potential for scaled deployment in controlled aquaculture or photobioreactor environments.

At larger spatial scales, UAV-based macroalgal monitoring has already proven effective for mapping coastal ecological change [23]. The high accuracy across taxa observed in bright-field microscopy suggests that similar CNN-based frameworks could be adapted for multi-scale monitoring workflows, bridging micro- and macroecological applications.

Taken together, the convergence of quantitative performance, species-level separability, and biologically interpretable visualizations demonstrates that modern CNN architectures—especially compound-scaled EfficientNets and deep residual networks—are highly capable of resolving morphological differences within Scenedesmaceae from bright-field microscopy alone. This alignment between biological expectation, computational behavior, and explainability strengthens the case for adopting explainable deep learning frameworks as trusted tools in ecological monitoring, water-quality analysis, biodiversity assessment, and microalgal biotechnology.

Finally, the observed consistency across all architectures tested is notable in light of recent systematic reviews emphasizing variability in deep learning performance depending on dataset composition, imaging modality, and taxonomic scope [16,33,34]. These reviews [34] further reinforce that robust cross-modal generalization—demonstrated here through high performance in simple bright-field conditions—is critical for the development of operational ecological AI systems.

## 5. Conclusions

This paper introduces an interpretable deep learning framework for the automated classification of three morphologically diverse microalgal species via bright-field microscopy. All assessed CNN architectures exhibited consistently high performance, indicating that dependable species-level differentiation may be attained under typical imaging settings when bolstered by a meticulously curated dataset and a cohesive preprocessing pipeline.

XAI methodologies, such as Grad-CAM and saliency mapping, offered enhanced understanding of the models’ decision-making processes. The visualizations emphasized morphologically significant areas while reducing the impact of background artifacts, validating that the classifiers depended on biologically relevant image characteristics. This interpretability enhances confidence in the framework and endorses its prospective application in ecological, environmental, and biotechnological fields.

The amalgamation of high-performing deep learning architectures with explainability methodologies creates a transparent and resilient framework for AI-assisted algae taxonomy. These systems offer considerable potential for ecological surveillance, biodiversity, and biomass-related biotechnologies. Subsequent research could enhance this paradigm by integrating larger and more diverse datasets, combining new imaging modalities such as fluorescence, hyperspectral, or holographic microscopy, and encompassing broader ecological contexts to provide scalable and adaptive tools for aquatic ecosystem intelligence.

## Figures and Tables

**Table 1:** Overview of the architectural features and computational intricacies of the deep learning models assessed in this research.

Model	Input Resolution	Parameters (Millions)	FLOPs (Billions)	Key Architectural Features
EfficientNetB0	224 × 224 × 3	5.3	0.39	Baseline model; compound scaling with mobile inverted bottleneck blocks
EfficientNetB1	240 × 240 × 3	7.8	0.70	Increased width and resolution for improved feature extraction
EfficientNetB2	260 × 260 × 3	9.2	1.00	Optimized for small datasets with enhanced receptive fields
EfficientNetB3	300 × 300 × 3	12.0	1.80	Balanced scaling across dimensions
EfficientNetB4	380 × 380 × 3	19.0	4.20	Higher input size; suitable for fine-grained image details
EfficientNetB5	456 × 456 × 3	30.0	9.90	Increased network depth and capacity
EfficientNetB6	528 × 528 × 3	43.0	19.00	Large-scale model for high-resolution datasets
EfficientNetB7	600 × 600 × 3	66.0	37.00	Deepest and most complex EfficientNet variant
DenseNet201	224 × 224 × 3	20.0	4.40	Dense connectivity between layers; efficient gradient propagation
ResNet152V2	224 × 224 × 3	60.0	11.30	Residual learning with identity mapping; stable deep network training
Xception	299 × 299 × 3	22.9	8.40	Depthwise separable convolutions; efficient spatial–channel decoupling
NASNetLarge	331 × 331 × 3	88.9	23.80	Automatically optimized via neural architecture search (NAS)

**Table 2:** Overall performance of tested CNN architectures in terms of training accuracy, validation accuracy, test accuracy, and loss metrics.

Model	Training Accuracy	Validation Accuracy	Test Accuracy	Validation Loss	Test Loss	Training Time (s)
EfficientNetB0	0.898	0.970	0.944	0.520	0.640	255
EfficientNetB1	0.897	0.916	0.903	0.468	0.526	368
EfficientNetB2	0.918	0.945	0.916	0.475	0.563	369
EfficientNetB3	0.894	0.949	0.899	0.550	0.683	374
EfficientNetB4	0.934	0.945	0.899	0.467	0.555	690
EfficientNetB5	0.953	0.945	0.916	0.422	0.477	1470
EfficientNetB6	0.928	0.941	0.899	0.535	0.704	973
EfficientNetB7	0.926	0.924	0.873	0.476	0.550	1906
DenseNet201	0.856	0.937	0.890	0.436	0.534	313
NASNetLarge	0.946	0.975	0.928	0.415	0.491	670
Xception	0.935	0.937	0.928	0.434	0.485	539
ResNet152V2	0.975	0.975	0.975	0.373	0.391	520

**Table 3:** Overall and class-wise performance metrics of tested architectures.

Model	Macro Precision	Macro Recall	Macro F1-Score
EfficientNetB0	0.934	0.916	0.915
EfficientNetB1	0.906	0.904	0.904
EfficientNetB2	0.928	0.916	0.916
EfficientNetB3	0.913	0.899	0.898
EfficientNetB4	0.916	0.899	0.898
EfficientNetB5	0.928	0.916	0.916
EfficientNetB6	0.916	0.899	0.898
EfficientNetB7	0.880	0.874	0.874
DenseNet201	0.908	0.891	0.889
NASNetLarge	0.942	0.928	0.928
Xception	0.933	0.929	0.929
ResNet152V2	0.976	0.975	0.975

## Data Availability

The datasets analyzed and/or generated during the current study are available from the corresponding author on reasonable request.

## Abbreviations

The following abbreviations are used in this manuscript:

XAI	Explainable Artificial Intelligence
Grad-CAM	Gradient-weighted Class Activation Mapping
CNNs	Convolutional Neural Networks
VGG	Visual Geometry Group
UAV	Unmanned Aerial Vehicle
CNN–SVM	Convolutional Neural Network-Support Vector Machine
CLAHE	Contrast-Limited Adaptive Histogram Equalization
NAS	Neural Architecture Search
FLOPs	Floating Point Operations Per Second
ROC	Receiver Operating Characteristic
AUC	Area Under The Curve
